# Cyclodextrins and Their Derivatives as Drug Stability Modifiers

**DOI:** 10.3390/ph16081074

**Published:** 2023-07-28

**Authors:** Virginia Aiassa, Claudia Garnero, Ariana Zoppi, Marcela R. Longhi

**Affiliations:** Unidad de Investigación y Desarrollo en Tecnología Farmacéutica, UNITEFA-CONICET, Facultad Ciencias Químicas, Departamento de Ciencias Farmacéuticas, Universidad Nacional de Córdoba, Córdoba 5000, Argentina; viraiassa@unc.edu.ar (V.A.); cgarnero@unc.edu.ar (C.G.)

**Keywords:** inclusion complexes, chemical stability, physical stability, stabilization

## Abstract

Cyclodextrins (CDs) are cyclic oligosaccharides that contain a relatively hydrophobic central cavity and a hydrophilic outer surface. They are widely used to form non-covalent inclusion complexes with many substances. Although such inclusion complexes typically exhibit higher aqueous solubility and chemical stability than pure drugs, it has been shown that CDs can promote the degradation of some drugs. This property of stabilizing certain drugs while destabilizing others can be explained by the type of CD used and the structure of the inclusion complex formed. In addition, the ability to form complexes of CDs can be improved through the addition of suitable auxiliary substances, forming multicomponent complexes. Therefore, it is important to evaluate the effect that binary and multicomponent complexes have on the chemical and physical stability of complexed drugs. The objective of this review is to summarize the studies on the stabilizing and destabilizing effects of complexes with CDs on drugs that exhibit stability problems.

## 1. Introduction

Stability is a key quality feature in pharmaceuticals. It influences the efficacy and safety of drugs, while providing the basis for the choices of manufacturing processes, formulations, packaging materials, and storage and transportation conditions [1]. The stability of pharmaceutical products is defined as their ability to maintain the established specifications of identity, potency, and purity within a specified period of time [2]. Due to the presence of labile groups in their molecular structure, some pharmaceutical compounds are susceptible to chemical breakdown under certain conditions. The main pathways by which a drug can be degraded include hydrolysis, dehydration, isomerization, racemization, oxidation, and photodegradation. In other cases, drugs undergo physical degradation changes, rather than chemical ones, which cause a range of alterations in their physical state. 

An example of physical instability is phase transformation [3]. Numerous active pharmaceutical ingredients (API) with unquestionable therapeutic value, due to their high intrinsic activity, may encounter significant limitations in clinical use because of inadequate chemical or physical stability characteristics. 

In this context, the application of pharmaceutical technology to drug stabilization is an interesting and demanding activity. The complex formation between drugs and excipients is a commonly used pharmaceutical strategy for modifying drug stability. Complexation can be defined as the reversible association of molecules of a substrate with those of a receptor to form a complex that shows a definite stoichiometry and physical–chemical properties, which can be substantially different from those of the compounds that formed it. 

**Figure 1:**
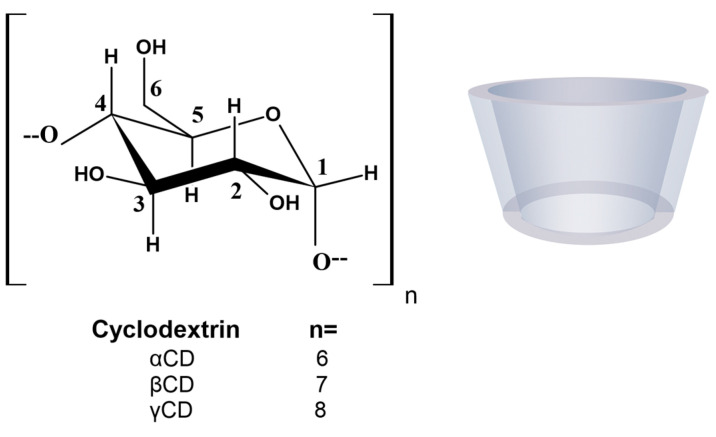
Molecular structure and schematic representation of the truncated cone shape of cyclodextrins.

Cyclodextrins (CDs) are versatile excipients that are widely used in the pharmaceutical industry as receptor molecules to form complexes with drugs. CDs are cyclic oligosaccharides composed of α-1,4-linked D-glucopyranoside subunits obtained through enzymatic starch degradation. In 1948, the composition of each of the native CDs was accurately defined when they were adequately purified and their crystal structure was resolved [4]. There are two kinds of CDs available: native CDs and modified CDs. The most common native CDs used for pharmaceutical applications are αCD, βCD, and γCD (Figure 1), consisting of 6, 7, and 8 glucose units, respectively, although larger CDs are known [5,6].

**Figure 2:**
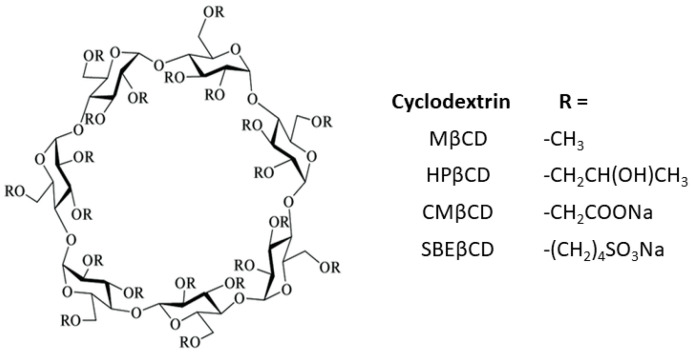
Schematic representation of substituted cyclodextrins.

In order to improve their solubility and their ability to enable complex formation, the native CDs have been modified. These derivatives are produced by adding diverse functional groups to the hydroxyl groups located in positions 2, 3, and 6 of the glucose molecule. Some of these groups are hydroxypropyl, methyl, carboxymethyl, and sulfobutylether β-cyclodextrins (HPβCD, MβCD, CMβCD, and SBEβCD) (Figure 2). CDs have the three-dimensional shape of a truncated cone, due to the ^4^C_1_ chair conformation of the glucopyranoside units [7]. The primary hydroxyl groups attached to carbon 6 of each glucopyranoside unit are located on one edge of the ring, while the secondary hydroxyl groups attached to carbons 2 and 3 are located on the other edge. The diameter of the cavity is larger at the edge of the secondary hydroxyls (the wide edge) than at the edge of the primary hydroxyls (the narrow edge), because free rotation of the latter reduces the effective diameter of the cavity. All of the hydrophilic groups are concentrated around the cavity openings and oriented outward, resulting in a highly hydrophilic external face. The cavity is lined by hydrogen atoms and glycosidic oxygen bridges, giving it a distinct hydrophobic and apolar character [8]. This dual property of having a hydrophilic exterior and a hydrophobic cavity means that these macromolecules can incorporate organic molecules inside, forming what are known as inclusion complexes. 

The ability of CDs to form inclusion complexes has led to a wide range of applications in the pharmaceutical industry. Complexation with CDs can be used to enhance the solubility, dissolution rate, permeability, absorption, bioavailability, physical state, volatility, partition coefficient, biological activity, and stability of many drugs. Therefore, there are marketed pharmaceutical formulations containing these oligosaccharides, such as those mentioned in Table 1. In terms of their ability to modify drug stability, although the inclusion complex sometimes has greater physical and chemical stability than the pure drug, it has been demonstrated that CDs can promote the degradation of other drugs [9,10,11]. The structure of the inclusion complex that is formed depends on the drug and the CDs used, which explains why certain drugs are stabilized while others are destabilized. Additionally, depending on the CDs employed for a drug’s complexation, the same drug may or may not be stabilized [9,12].

Although the formation of binary complexes between CDs and drugs with degradation problems is an effective strategy for improving stability, in some cases CDs have a low complex efficiency (CE) toward the guest drug, requiring a high concentration of CDs to form inclusion complexes. By combining a ternary agent with a binary complex to create a multicomponent complex, it is possible to raise the stability constant and the CE values, due to the synergistic interaction between these components [13,14,15]. However, adding a third component that competes with the drug for inclusion in the CD cavity can lower the overall efficiency of the multicomponent system. Therefore, the selection of adequate ternary compounds is a crucial step in the development of a supramolecular system. The ability of auxiliary agents to interact with guest and host molecules, and even with drug–CD complexes, determines which one will be most suitable for forming a multicomponent complex. Numerous substances have been identified as potential auxiliary agents. In particular, aminoacids, polymers, organic bases, and hydroxyl agents have received the most attention [16]. The analysis of thermodynamic parameters reveals that different forces and interaction mechanisms contribute to the development of complexes in the presence of different auxiliary agents. Given that each binary complex would be improved by a certain third agent, molecular modeling techniques emerge as a very potent screening strategy to choose the best adjuvant, enabling the carrying out of massive experimental screenings. It is possible to rationally develop an appropriate complex by taking into account the structural, dynamic, and energetic data of a collection of components [17,18]. In addition, interaction and/or inclusion of drug labile groups in the supramolecular complex is required for improving drug stability.

The formation of complexes with CDs has become common in order to improve the therapeutic applicability of different drugs [16]. In considering the ability of CDs to stabilize certain drugs while destabilizing others, which is attributed to the different structures of the inclusion complexes formed, it is necessary to study the effects of complexation on the stability of the included drugs. Therefore, the main objective of this review is to describe the impact of CDs on the chemical and physical stability of drugs. Table 2 summarizes the CD complexes described in the literature that was considered.

## 2. Enhancement of Drug Stability Induced by CDs

### 2.1. Biological Products

Given the increasing use of protein-based drugs, controlling the physical stability of proteins is crucial for the pharmaceutical sector. Excipients are typically used in the formulation process to aid in the stability of biopharmaceuticals. Promising excipients are commonly found in protein-based medicines that are chosen for their ability to stabilize specific properties, including the preservation of natural bioactive conformations [66]. Although the effects that CDs may have on the stability of some proteins have been studied, the mechanisms by which stabilization occurs are not clear. To better understand the mechanism by which CDs stabilize proteins, Samra et al. [19] examined the effects of different types of natural and modified CDs on the physical stability of three proteins of pharmaceutical relevance [*Clostridium difficile* Toxoid A, V antigen (LcrV) or *Yersinia pestis* low-calcium-response V, and fibroblast growth factor 10 (FGF-10)]. Changes in proteins’ tertiary and secondary structure and aggregation behavior as a function of pH and temperature in the presence of various CDs were monitored. The kind of CD present and the different groups substituted on the CD are likely to have an impact on the effects of the CD on both the structural alterations and the aggregation reported in this work. It was observed that, in general, all the CDs studied produced inhibition of protein aggregation. Although the mechanism by which the degree of substitution influences protein stability is unknown, the results may indicate that, in some cases, small changes in the degree of substitution can influence the stabilizing effect of a given CD on a specific protein. The fact that little correlation is detected between the changes in the aggregation behavior and the simultaneous stabilization of the structure of the protein suggests the occurrence of more than one stabilization mechanism in the presence of CDs. 

Otzen et al. [20] reported that the aggregation of human growth hormone can be inhibited using various natural and modified CDs. The authors demonstrated that CDs bind to the aromatic side chains and lead to a more unfolded conformation of human growth hormone at low pH, preventing aggregation. The effect of CDs on protein stability is dependent on the derivative, the molar substitution, and the concentration of CDs. Some βCD derivatives were more effective in preventing the aggregation of this protein than α and γCD, which can be attributed to the different dimensions of the cavity. Substituents on the CD ring also influence protein aggregation; for example, glycosyl- and maltosyl-βCD have a stronger stabilizer effect than HPβCD and SBEβCD, probably because they are only substituted at C6 and provide the aromatic side chain easier access to the CD cavity.

Monoclonal antibodies are another important category of biological drugs. These formulations are administered mainly parenterally. Although recent discoveries suggest the pulmonary route as a possible alternative for local and/or systemic delivery of proteins and/or peptides, there are numerous obstacles in the formulation of inhalable proteins due to their intrinsic instability. As a result, dry powder formulations are suggested to improve product stability. Spray drying can produce particulate systems with size ranges from 1–5 μm, appropriate for inhalation [21]. In this process, an appropriate design of the formulation is critical in minimizing mechanical and thermal stresses during processing. Different excipients, including CDs, are used to improve particle properties and protein stability. 

Ramezani et al. [21] investigated the effects of HPβCD and βCD on the stability and particle properties of spray-dried IgG for the development of inhalable protein formulations. In that study, it was determined that both the type of excipient and its proportions influenced the stability of the antibody. HPβCD conserved the protein secondary structure and inhibited aggregation during spray drying, probably due to the fact HPβCD is a hydrophilic CD with surface active properties. The use of βCD, on the other hand, led to the development of larger particles.

Glucagon is a peptide hormone used for the treatment of hypoglycemia; due to its poor solubility, it is administered as an acidic aqueous injection. However, this hormone shows chemical and physical degradation in acidic solutions. In this sense, the interactions between glucagon and γCD molecules in inclusion complexes were evaluated by Matilainen et al. [22] to investigate the effect on chemical and physical stability. The presence of γCD improved the chemical half-life of glucagon at pH 2.0 and extended the lag-time before aggregation at pH 2.5. Nuclear magnetic resonance (NMR) studies showed that the side chains of the aromatic amino acid residues (Phe6, Tyr10, Trp25, Tyr13, and Phe22) and leucine (Leu14 and Leu26) of glucagon interacted with the cavity of the γCD molecules. This indicated that glucagon forms inclusion complexes with γCD in acidic solutions, resulting in an enhancement in its physical and chemical stability. With respect to physical stability, all of the aromatic amino acids were included in the γCD cavity, according to NMR data. This provides concrete evidence that the intermolecular hydrophobic interactions between the side chains of aromatic amino acids (Tyr, Phe, and Trp) may begin the glucagon aggregation and that γCD reduces the aggregation by encapsulating these hydrophobic residues into the inclusion complexes. Regarding chemical stability, it was observed that the N-terminal neighbor of Asp 15 (Leu14) and the C-terminal neighbors of Asp 9 (Tyr10) and Asp 21 (Phe22) of glucagon formed inclusion complexes with γCD. Therefore, it is possible that the steric barrier caused by γCD complexation can be used to explain how CDs stabilize the drug against chemical degradation [22].

Insulin glargine is an analogue of insulin that has a prolonged duration of action; it is used to treat diabetes. It is supplied in an acidic solution that is neutralized at the injection site, resulting in the formation of microprecipitates subcutaneously after injection, after which insulin glargine is gradually released into circulation. Furthermore, proteinase, such as trypsin, cleave insulin glargine at the carboxyl side at the site of injection and in the systemic circulation. Uehata et al. [23] demonstrate that the formation of a complex with SBEβCD reduced its enzymatic degradation at the injection site by impeding tryptic cleavage of insulin glargine by trypsin and increased the dissolution rate of insulin glargine from its precipitates, which improved both bioavailability and the persistence of the sustained-glucose-reducing effect. It is hypothesized that the insulin glargine/SBEβCD complex will decrease the interaction between the negatively charged aspartic acid in the trypsin catalytic region and the positively charged lysine and/or arginine in insulin. SBEβCD contains negative charges originating from sulfobutyl groups that can interact with these positively charged residues of insulin.

### 2.2. Herbal Compounds

Natural products have a variety of biological and pharmacological effects, making them suitable for use in the development of therapeutic formulations with medicinal applications. In addition, natural products, as well as their derivatives, have been crucial instruments for the identification of novel targets such as receptors. However, some of them have numerous disadvantages, such as poor oral absorption and low storage stability, resulting in restricted usage and efficacy. Formulation studies involving natural materials using complexation with CDs to improve their properties have received considerable attention, resulting in improved products with better storage stability. For example, Z-ligustilide is a natural benzoquinone derivative found in many widely used Chinese herbal medicines. Z-ligustilide has a wide range of pharmacological properties, including antiinflammatory and neuroprotective effects, but shows chemical instability. Lu et al. demonstrated that HPβCD increases the photostability of Z-ligustilide. After being stored under light for 6 days, the degradation of free and complex Z-ligustilide was 77.9% and 22.2%, respectively [24]. 

Resveratrol, a natural stilbene found in various vegetables and fruits, has been suggested as an emerging alternative, with significant pharmacological effects due to its anti-inflammatory, antioxidant, proapoptotic, and anti-cancer properties. However, resveratrol has low chemical stability in neutral and basic media due to hydrolysis, which limits its therapeutic uses. According to Wang et al. [25], resveratrol stability improved significantly after complexation with SBEβCD as a result of encapsulating inside a tube-like structure created by the CDs, where the hydroxyl groups of the resveratrol establish hydrogen bonds with the CDs. In stability studies conducted at physiological pH 7.4, this complex demonstrated a 27-fold increase in drug levels that remained after 8 days. Additionally, the resveratrol:SBEβCD complex inhibited the drug degradation kinetics in biological matrices. After 96 h of incubation, it was found to protect against drug hydrolysis in biologic plasma (rat and human), with a 4-fold increase in calculated half-life. As a result, resveratrol:SBEβCD demonstrated potential for pulmonary delivery in the inhalable therapy of lung cancer, with substantially improved stability when compared with resveratrol. 

Moreover, the oxyresveratrol:HPβCD complex studies revealed similar stabilizing results associated with the inclusion of the drug molecule in the cavity of the CDs. Oxyresveratrol, which is frequently present in *Moraceae plants*, is a natural tyrosinase inhibitor that is unstable when exposed to light and heat. Additionally, prooxidant agents can quickly oxidize it, or it can be converted into a cis-structure with minimal biological activity. As storage time increased, the oxyresveratrol content significantly decreased, and the color of the sample deepened, possibly as a result of oxidation. However, after 30 days of storage with light at a range of 4–50 °C, the oxyresveratrol:HPβCD complex successfully protected it against degradation, with stronger temperature stability [27]. 

Carlotti et al. reported that the inclusion complex of quercetin, a flavonoid antioxidant, in α and βCD reduced photochemical reactivity and helped to improve its solubility [28]. In addition, quercetin and resveratrol are two drugs that exhibit antioxidant and anti-inflammatory properties that are necessary to control dry eye disease, but due to their low aqueous solubility and low chemical stability, their application as topical eye drops is restricted. To overcome these drawbacks, Krstic et al. [26] carried out a study in which they compared binary complexes with quercetin, resveratrol, and HPβCD and multicomponent complexes with the addition of hyaluronic acid on the increase in the solubility and stability of both drugs. The chemical stability of quercetin and resveratrol was determined in a pH 7.4 phosphate buffered solution (PBS) at 25 °C. Degradation half-lives of both compounds were prolonged for complexes with HPβCD and, although the multicomponent complex with HPβCD and hyaluronic acid was shown to improve drug stability, it was found that this improvement was dependent on the concentration of the polysaccharide, since 0.1% *w*/*v* hyaluronic acid produced the highest stabilization for both polyphenolic compounds. In addition, in in vitro research, none of the complexes showed a cytotoxic effect in studies on corneal and conjunctival cell lines.

According to Savic et al., complexing rutin with βCD and HPβCD improved rutin photostability by 2.5 and 5.4 times, respectively, under UVB radiation exposure [29]. The stabilizing effects of dimethyl-β-cyclodextrin (DMβCD) were recently reported. The ethanol extract of *Cannabis sativa*, which is easily degraded by high temperatures and prolonged storage, showed greater stability after being incorporated into the DMβCD cavity [30].

### 2.3. Cosmetic Products

Sunscreens are topical formulations that protect skin against ultraviolet radiation (UVR). They have active ingredients such as UV filters that absorb, reflect, and/or scatter UVR. Nevertheless, UV filters are capable of degradation upon exposure to oxygen and sunlight. CDs can protect the encapsulated molecules of UV filters, promoting their stability against oxidative stress and external stimuli [67]. For example, a significant photostability increase of UV filters (oxybenzone, octocrylene, and ethylhexyl-methoxycinnamate) was registered when βCD was used in lotion formulations, compared to those without βCD [31]. 

Phenylbenzimidazole sulfonic acid is an UV-B filter authorized for use as a sunscreen agent in cosmetic products to prevent skin erythema and DNA mutagenic photolesions. However, it has been reported that when exposed to UV radiation, this agent can photodegrade or act as a photosensitizer, generating a mixture of free radicals and active oxygen species that cause photoinduced DNA damage [68]. Scalia et al. [32] demonstrated that complexation with HPβCD is more effective than with randomly methylated β-cyclodextrin (RMβCD) in increasing sunscreen stability at acidic pH conditions that are ideal for topical administration. The photo-induced sunscreen decomposition was not severely impacted by complexation with RMβCD, whereas the HPβCD use resulted in a significant reduction of photogedration. Moreover, the photostabilizing effects of HPβCD were maintained for 6 months. Furthermore, the HPβCD complex had no important impacts on the photoprotective capacity and the degree of UV absorption of this agent. These results indicate that the complexation of phenylbenzimidazole sulfonic acid with HPβCD by incorporation into the CD cavity is a useful strategy for improving sunscreen photostability and reducing the light-induced generation of free radicals. Consequently, this CD complex should potentially decrease the damage produced by sunscreen.

Tretinoin, or all-trans retinoic acid, is effective for treating different skin disorders by topical application, but it is a photolabile compound. Caddeo et al. [33] compared the stability of free tretinoin in methanol and their complex with βCD and determined that the complex showed a clear increase in drug stability under exposure to both fluorescence and UV light.

### 2.4. Photodynamic Therapy Drugs

The principle of photodynamic therapy is to combine a photosensitizer, which is a photoactivatable molecule, with light, generally in the visible spectrum, to produce cytotoxic reactive oxygen species (ROS) [69]. Cancer treatment with photodynamic therapy demands the administration of a photosensitizer followed by visible light excitation. The excited photosensitizer produces ROS, leading to cellular damage or death [70]. Nevertheless, the non-targeting capability and the side effects of photodynamic therapy, as well as the low stability and solubility of a photosensitizer, limit its further development. 

Recently, supramolecular chemistry based on host–guest interaction with CDs has attracted attention in overcoming the limitations of these therapeutic agents [71]. Lu et al. studied and compared complexes based on tetra-1,2-diethylamino substituted zinc (II) phthalocyanine with αCD, βCD, and γCD and their interaction modes of inclusion, potential to reduce their aggregation degree, ROS generation ability, and in vitro anticancer activities. The results showed that the aggregation degree of phthalocyanines was considerably decreased and the photodynamic activity and water solubility were increased for the host–guest inclusion complexes. In particular, they discovered that the interaction modes between phthalocyanines and CDs changed with different kinds of CDs. The complex of phthalocyanines and βCD showed superior stability, because they could establish more hydrogen bonds than the complexes with α and γCDs [34].

### 2.5. Synthetic APIs

In these times of great advances in science and technology, a large number of interventions are being implemented to reduce health-related diseases. Within these actions, organic synthesis stands out. It contributes to the development of effective drugs for their application in therapeutic medicine. Some of these drugs have stability problems that can be overcome using CDs.

**Figure 3:**
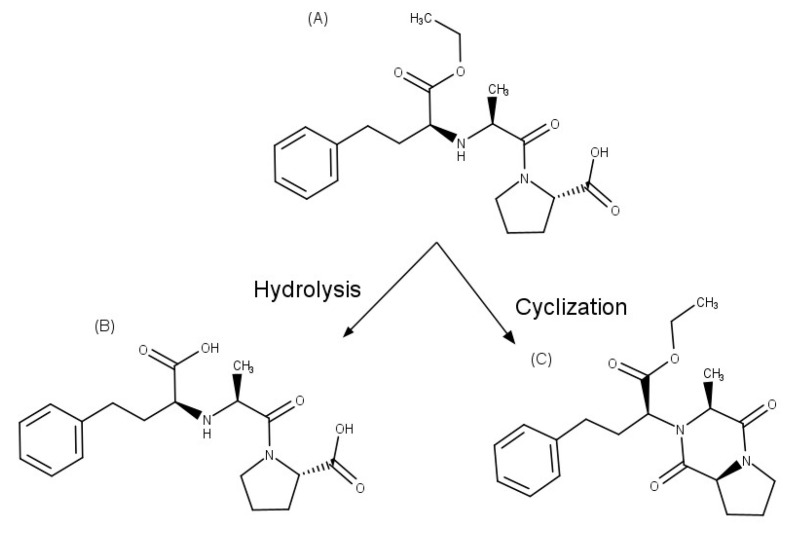
Chemical structures of: (**A**) enalapril, (**B**) enalaprilat, and (**C**) enalapril diketopiperazine.

Enalapril, an inhibitor of the angiotensin-converting enzyme that is used to reduce high blood pressure and to prevent or treat heart failure. This API is degraded to enalaprilat by hydrolysis in solutions with a pH of 5 or above, while diketopiperazine is the main degradation product formed in the solid state and by photodegradation in solution (Figure 3). Zoppi et al. [35,36] investigated the effect of complex formation with βCD on the stability of this drug. Since enalapril undergoes solid-state degradation due to interaction with some excipients used in tablet formulation, the stability of this API was examined in the presence of magnesium stearate in solid state after six months of storage at 40 °C and 75% relative humidity, using an HPLC stability-indicating method. In these studies, enalapril complexed with βCD proved to be 2.1 times more stable than the drug alone in solution. In addition, enalapril cyclization, which generates enalapril diketopiperazine in the presence of excipients such as magnesium stearate in the solid state, was prevented by the complex formation with βCD, allowing a drug recovery of 95% and 82% for the complex and pure drug, respectively.

Hydrocortisone is a corticosteroid drug that is widely used in therapy, due to its anti-inflammatory and immunosuppressive properties. With the aim of formulating a stable topical ophthalmic solution of the drug, Davies et al. [37] studied the effect of HPβCD on its chemical stability. In the chemical stability studies, it was determined that the decomposition of the drug in aqueous solutions of PBS pH 7.4, both in the absence and in the presence of various concentrations of HPβCD, followed pseudo-first-order kinetics. The samples were analyzed by a stability-indicating HPLC assay, and it was found that the hydrolysis rate constant decreased as the percentage of complexed drug increased. Thus, it was postulated that the inclusion of the hydrocortisone molecule inside the hydrophobic cavity of these CDs provides some degree of protection against hydrolysis. 

The stabilizing effect of HPβCD differs from that reported by Andersen and Bundgaard [39] for the native βCD, since they determined that the complexation with the βCD did not present any stabilizing effect on the decomposition of hydrocortisone in neutral or acidic aqueous solutions, as well as that the presence of βCD accelerated decomposition under alkaline conditions. Therefore, Davies et al. [37] proposed that the stabilizing effect observed in their study may be the result of the extension of the hydrophobic cavity of the βCD by the hydroxyalkylation of the primary and secondary hydroxyl groups, favoring the inclusion and, consequently, the protection of the C17 side-chain, which is the primary pathway for hydrocortisone degradation in solution. In addition, El Maghraby and Alomrani [38] determined that hydrocortisone complexation with HPβCD in solution significantly increased drug stability after gamma irradiation. As a result, the HPβCD incorporation into hydrocortisone aqueous solutions significantly improved drug stability and had the potential for preparing eye drops that required sterilization.

Famotidine is an H2 histamine receptor antagonist that is used as an antiulcer agent. It is susceptible to acid-catalyzed hydrolysis. HPβCD [40] and CMβCD both showed pronounced stabilizing effects on the acidic degradation of famotidine solutions; however, SBEβCD [42] induced a destabilizing effect. The different ways that famotidine interacts with these CDs were found to be responsible for this particular behavior, with the drug being positioned in a more hydrophobic environment when CMβCD was used. Furthermore, the polymorphic transition of famotidine affects its bioavailability and therapeutic efficacy. This drug exists in two solid forms: the more stable form A and the more soluble and metastable form B that is used as an API in formulations. However, the transformation of form B to form A is caused by production processes and/or storage conditions. Jamrógiewicz et al. [41] found that the physical stability of famotidine is improved by HPβCD, CMβCD, and SBEβCD. The supramolecular systems formed with these CDs demonstrated inhibition of the polymorphic transformation of famotidine during compression.

Lansoprazole, which is a proton pump inhibitor used to treat gastric and duodenal ulcers, is susceptible to chemical degradation due to its sensitivity to low pH, light, heat, and humidity. The sulfinyl group is the most fragile group in the lansoprazole structure and is the determining factor for its stability. Therefore, the inclusion of this group in the CDs cavity would have protective effects. Notable differences were evidenced in the lansoprazole photostability between HPβCD and βCD complexes, which were attributed to the fact that the sulfinyl group of the drug was in the cavity of HPβCD but not in the βCD [43].

Camptothecin, a very effective drug with broad-spectrum activity against several types of cancer, is rapidly converted to the corresponding water-soluble and pharmacologically inactive carboxylate form in aqueous buffer solutions (pH > 7), which restricts its therapeutic potential. Kang et al. [44] found that camptothecin hydrolyzes more slowly in PBS (pH 7.4) when it is encapsulated in randomly substituted dimethyl-β-cyclodextrin (RDMβCD) than when it is in its free form. A 10-fold increment in camptothecin stability was observed by the addition of RDMβCD at a 25% *w*/*v* concentration. 

In addition, nintedanib is a kinase inhibitor and antifibrotic drug that has been approved to treat idiopathic pulmonary fibrosis, as well as a variety of cancers. Its oral bioavailability was reported to be approximately 5%. A study was conducted with the aim of increasing the stability of the API in the intestinal environment and improving its intestinal permeability and, therefore, its bioavailability [45]. It was determined that the stability of nintedanib in PBS and simulated intestinal fluid was enhanced by the complexation with SBEβCD. In addition, the bioavailability of the drug could be improved by enhancing its stability in simulated intestinal fluid and PBS.

The posaconazole drug is a potent antifungal that is susceptible to oxidation [72]. In the presence of βCD, the drug’s stability was increased. Through computational simulation, it was observed that βCD acts as a posaconazole protecting agent, since it protects the portion of the molecule that is susceptible to oxidative attack, maintaining its complexation over time [46].

The photostability of nicardipine under exposure to UV (A)–UV (B) radiations in aqueous solution with different CDs was investigated [47]. A photoprotective effect was observed by βCD, HPαCD, and 2-hydroxyethyl-β-cyclodextrin, whereas γCD, MβCD, HPβCD, and HPγCD did not affect the nicardipine photostability. In contrast, αCD was found to promote drug photodegradation. Moreover, stereoselective photodegradation of rac-nicardipine was detected for the βCD complex with two different photodegradation profiles, with distinct kinetic constants for the two enantiomers. In addition, doxycycline hyclate, an active tetracycline class drug used as a broad-spectrum antibiotic, is another drug that has photostability problems. Complexation with βCD was reported by Kogawa et al. [48] to be a successful strategy for enhancing drug stability. After 6 h of exposure to UV light, pure doxycycline hyclate showed faster photodegradation than the complex form of the drug, with recovery percentages of 68% and 98%, respectively. 

Oxytetracycline hydrochloride is another broad-spectrum polymorphic tetracycline antibiotic with instability problems, including photodegradation, oxidative degradation, and hydrolysis. Bueno et al. [49] surprisingly observed that binary systems obtained with βCD only demonstrated a reduction in the degradation rate of oxytetracycline hydrochloride form III in the aqueous solution, with an increase of 20 h in the half time when compared to free form. This revealed distinct interactions between the CD and each drug polymorph, which influence their stabilizing effects. Moreover, doxorubicin, which is an anthracycline antibiotic used as a chemotherapeutic agent for treating solid tumors, is sensitive to light because of its chemical structure as a tetracyclic anthraquinoid aglycone. The doxorubicin photostability in aqueous solution was enhanced by its inclusion into the HPβCD cavity, with a degradation rate three times slower than that of the drug’s free form [50].

The multicomponent complex formation has demonstrated influence on the stability of drugs. Furosemide, which has an aminosulfonyl group in its structure, is a loop diuretic drug used in the treatment of arterial hypertension and edematous states. The effect of complaxetion with βCD and triethanolamine on the drug photodegradation processes was studied by Abraham-Miranda et al. [51]. It was possible to verify a stabilizing effect on the chemical degradation of furosemide when combining triethanolamine with βCD, showing the multicomponent complex stabilization parameters to be higher than those obtained with binary ones. Through the analysis of the XRPD patterns and the NMR spectra in the solid state, obtained for furosemide and the multicomponent system after 6 months of storage, the physical stability of the samples and the absence of polymorphic transformations were verified.

**Figure 4:**
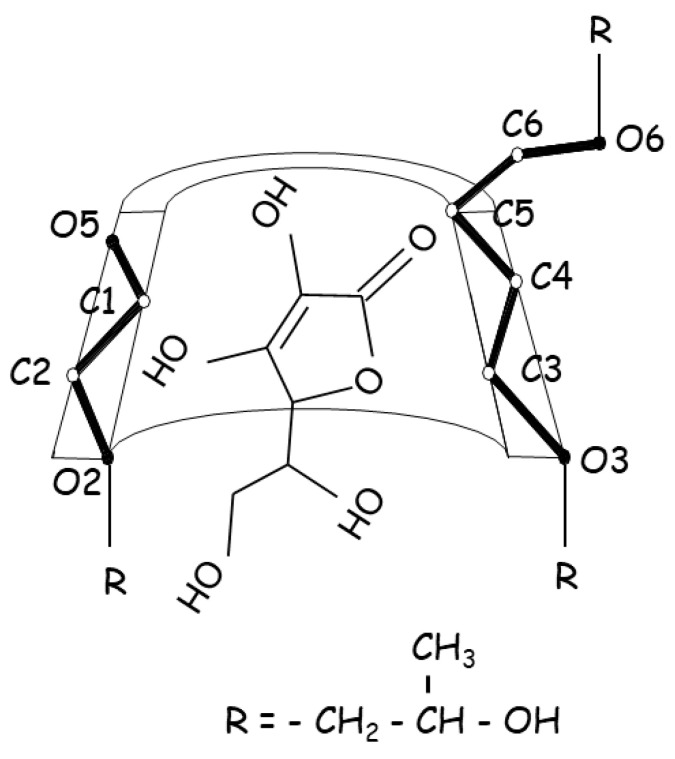
Proposed structure for the ascorbic acid:HPβCD inclusion complex associated with stabilizing effects.

Ascorbic acid, commonly known as vitamin C, acts as an antioxidant in the biological system, with recent studies showing significant antibacterial activity at acidic pH against Gram-positive and Gram-negative bacteria [73]. Ascorbic acid is widely employed as an antioxidant excipient in the pharmaceutical industry; however, it is particularly vulnerable to degradation in aqueous solutions because it has a lactone moiety, which is easily oxidized to dehydroascorbic acid and, thereafter, degraded. Ascorbic acid was complexed with HPβCD to improve aqueous stability by inhibiting oxidative degradation in aqueous solutions (Figure 4) [52]. In the presence of 1% HPβCD, its stability was increased 3-fold, which resulted in an increase in ascorbic acid half-life in aqueous solution from 3.4 h to 10.0 h. However, ascorbic acid was stabilized by HPβCD in the acidic pH region, while it was destabilized in the alkaline pH zone, indicating that the stabilizing effect of HPβCD is pH-dependent. The unionized form of ascorbic acid in acid solution, which interacted more strongly with the cavity than its ionized form, was related to the stabilizing effects, while in alkaline solution, the degradation was increased in the presence of CDs, suggesting that the catalytic combined effect was synergistic (i.e., a greater acceleration was achieved when alkaline media and CDs were used together), inferring that this process was facilitated by the interaction of the ester part of the ascorbic acid with the hydroxyl groups of the CDs, which appeared as alkoxide ions in this pH zone. 

In addition, the complexation of ascorbic acid with HPβCD in the presence of triethanolamine was studied, and the incidence of this multicomponent complex on the stability of this vitamin was determined. The results revealed that the protection against degrading factors was notably higher for the multicomponent complexes. 

The effect of this multicomponent complex on the photostability of ascorbic acid exposed to artificial and diffuse daylight was also investigated by Garnero et al. [53]. The results showed that the complexes markedly decreased photodegradation, with an 11- and 35-fold increase in photostability of ascorbic acid, depending on the ligand concentration and the irradiation source, emphasizing that the multicomponent complex produced superior results for stabilization against light exposure than triethanolamine alone. The results obtained in these studies, which allowed a significant improvement in stability in aqueous solutions and in the photostability of ascorbic acid, due to the formation of multicomponent complexes, enabled the development and validation of methods by UV spectrophotometry and HPLC for the measurement of this water-soluble vitamin in different pharmaceutical formulations. 

In addition, Saokham et al. [54] studied the protection capacity of γCD for ascorbic acid through the formation of multicomponent complexes with the addition of polyvinyl alcohol. Based on the results, they determined that the complex formation with γCD and polyvinyl alcohol was able to protect ascorbic acid from the oxidation reaction in aqueous solutions when the optimal concentrations of γCD and polyvinyl alcohol were used. 

Wang et al. [55] demonstrated that the stability of dihydroartemisinin at pH 7.4 Hank’s balanced salt solution can be improved by obtaining a multicomponent complex with HPβCD and soybean lecithin. The stability studies were performed at 37 °C, and dihydroartemisinin concentration was measured by HPLC. It was determined that the degradation follows first-order kinetics. The multicomponent complex showed a significant improvement in the stability of dihydroartemisinin, compared to the binary complex, with k values of 0.48, 0.38, and 0.13 h^−1^, for the pure drug and the binary and multicomponent complexes, respectively.

## 3. Degradation of Drugs Induced by CDs

Although complexation with CDs is one strategy for improving unfavorable properties, it was determined that the substitution of the hydroxyl groups on the CD molecules influences whether the API is destabilized or stabilized. Benzylpenicillin, a natural β-lactam antibiotic, is unstable in aqueous media, has low oral bioavailability, and has a short biological half-life. Furthermore, its degradation products can induce allergic reactions. Popielec [56] determined that HPβCD and RMβCD complexes reduced the benzylpenicillin hydrolysis in acidic conditions (pH 1.2 to 4.6), because the uncharged penem ring, which dominates at low acidic pH, has greater affinity for the hydrophobic CD cavity and the β-lactam ring of the API can be protected. However, the HPβCD complex increased the rate of API hydrolysis in neutral to basic media (pH 7.4 and 9.6) as a result of the charged penem ring that predominates at these pH values, which has a lower affinity for the CD cavity, accelerating the hydrolytic cleavage of the ring structure by the nucleophilic attack of the hydroxyl ion. 

In contrast, some stabilizing effect was observed for the RMβCD complex in the basic solution. Because RMβCD is more lipophilic and contains significantly fewer hydroxyl groups (i.e., around 60%) than HPβCD, it exhibited a higher stabilizing effect in acidic solutions and a less catalytic effect in basic solutions on the benzylpenicillin hydrolysis. Methylation of the hydroxyl groups decreases its capacity to form hydrogen bonds with β-lactam, which should eliminate the catalyzing effect, resulting in RMβCD being less destabilizing than HPβCD at pH 7.4 and even having some stabilizing effect at pH 9.6, where HPβCD increased the specific base cleavage of the β-lactam ring. In addition, these studies demonstrated that the accelerating effect was dependent on both the charge of the CD molecule and the number of accessible groups available. Therefore, the catalytic effect of γCD on the benzylpenicillin hydrolysis was enhanced by substitution of the hydroxyl groups by quaternary ammonium, sulfobutylether, or hydroxypropyl groups, whereas it was decreased by methylation and carboxylation. 

In the same way, in subsequent studies, Popielec [57] revealed the impact of the degree of methylation of the native CD molecules on the chemical stability of benzylpenicillin under physiological conditions, and determined that enhancing the concentration of CDs derivatives accelerated the degradation rate of benzylpenicillin. However, increased methylation of the hydroxyl groups at the CDs rim decreased the drug degradation, due to reducing or suppressing the CD catalyzing effect. Thus, the methylated γCD derivatives, randomly methylated-γCD and octakis(2,3,6-triO-methyl)-γCD, showed a 3-fold increase in benzylpenicillin stability in solution, compared with native CD. On the other hand, complete substitution of hydroxyl groups in heptakis(2,3,6-tri-O-methyl)-βCD not only prevented the catalytic effect, but additionally caused the protection of the fragile benzylpenicillin molecule within the complex. While the catalytic effect of native βCD is only slightly decreased by partial methylation in RMβCD and heptakis(2,6-di-O-methyl)-βCD. 

In addition, Loftsson and Johannesson [58] determined that the degradation of β-lactam antibiotics was accelerated in the presence of βCD and that the rate of degradation increases as the concentration of this native CDs is enhanced. Cefixime, a third-generation cephalosporin antibiotic, is susceptible to hydrolytic breakdown when reconstituting with water, but stable in the dry state. Cephalosporin antibiotics have a molecular structure that is remarkably similar to penicillin antibiotics, which are susceptible to hydrolysis at the lactam ring. Cefixime is, therefore, typically prepared as a dry solution or a tablet for oral administration to treat infections of the skin, the respiratory system, the urinary tract, and the skin structure. Pamudjia et al. [59] assessed the impact of βCD on the stability of cefixime in the liquid suspension dosage form. The rate of cefixime degradation was accelerated by the preparation of the cefixime:βCD inclusion complex by the freeze-drying method. On the other hand, when the inclusion complex was prepared by the kneading method, the rate constant for cefixime degradation was remarkably similar to that of the untreated pure drug and its physical mixture [59]. Therefore, as observed with other drugs, that study determined that obtaining the cefixime:βCD complex was not effective in achieving an increase in the stability of the API. 

Dan Córdoba et al. [60] studied the stability of rifampicin, another antimicrobial type, in binary and multicomponent complexes with γCD and γCD:arginine, respectively. Drug stability was reduced in the case of the RIF:γCD complex. This negative effect on drug stability was avoided by forming a multicomponent complex with arginine, which was shown not to affect drug stability. Furthermore, norfloxacin is a second-generation fluoroquinolone with poor biological availability associated with low absorption. The complex formation with CDs is a strategy that is widely used to solve this problem. However, the complexation of norfloxacin polymorphs with βCD had diverse impacts on the polymorphs’ stability, confirming that the interactions between each norfloxacin polymorph and βCD generate complexes with specific properties. Solid complexes of norfloxacin C and norfloxacin B hydrate with βCD, prepared in equimolar ratios by the kneading method and stored for 6 months under accelerated storage conditions, showed considerably different effects on stability. While the CD complex increased the photochemical stability of norfloxacin C without affecting its physical stability [61], a destabilizing effect was evidenced for norfloxacin B hydrate [62]. This effect of the complex was attributed to the moderated hygroscopicity of the solid system, which accelerated the chemical reactivity of the polymorph.

As previously mentioned, CDs sometimes have a destabilizing effect on some molecules. For example, omeprazole is a proton pump inhibitor that is frequently used to prevent and treat peptic ulcers, as well as to relieve the symptoms of gastro-esophageal reflux, which is very unstable, particularly in acidic aqueous solutions. βCD, DMβCD, HPβCD, and maltosyl-βCD (MaβCD) accelerated the omeprazole degradation, according to El-Badry et al. [63]. In t comparative study, the order of the effect determined for these CDs was βCD > DMβCD > MaβCD > HPβCD. It was observed that the native βCD has a greater negative effect on drug hydrolysis than the different derivatives. This result can be attributed to structural differences between βCD and its derivatives, since the βCD has free hydroxyl groups that can accelerate drug degradation. This finding was in agreement with that reported by Hiraayama et al. [64], who determined the negative effect that βCD had on the stability of prostaglandins, while its derivative, the DMβCD, showed a positive effect. 

Jansook et al. [65] investigated the addition of various organic salts to improve drug solubilization of irbesartan and candesartan cilexetil with γCD. Of these, Tris was the one that allowed better results, followed by sodium acetate, since it significantly increased the γCD solubilization of irbesartan and candesartan cilexetil. However, it was determined that at high salt concentrations, with which greater solubility of irbesartan and candesartan was achieved, chemical instability was produced. The balance between the drug’s ideal solubility and its chemical stability in aqueous solutions must, therefore, be taken into account when developing systems that can be used to establsih formulations. Stability studies were carried out in accordance with the International Conference on Harmonization (ICH) guidelines [74], for which the samples were stored in hermetically sealed glass vials under long-term and accelerated conditions. When comparing the chemical stability of aqueous solutions containing irbesartan or candesartan with γCD and organic salts, it was observed that the Tris salt complexes showed greater susceptibility than those of sodium acetate. This difference can be interpreted as the greater ionization of irbesartan and candesartan by the Tris salts, which leads to greater degradation. Therefore, to reverse this instability problem that occurs when both organic salts are present in high concentrations, the authors propose the conversion of these aqueous multicomponent complexes into solid complexes for reconstitution, in order to increase the shelf life of pharmaceutical products.

## 4. Conclusions

This review has collected data showing how complexation with CDs can modify drug stability. The analysis of the reviewed publications clearly demonstrated that the degree of drug stabilization or destabilization seen after complexation is influenced by the complexation pathway between the API and the CDs, as well as by the mechanism of API degradation. It is possible to conclude that CDs are an efficient approach to improving the chemical and physical stability of many APIs in solution and in solid state. It is clear, however, that when CDs were utilized as stabilizing agents, positive findings were not always attained, since this stability relied on a variety of factors, including the nature of the molecules studied as well as their orientation within the host cavity, the type and degree of substitution of the CDs, and the reaction medium. To obtain the desired impact on drug stability, it is essential to investigate which CDs produce the most stabilizing effect and which concentrations are most suited for each individual case. In addition, it is important to keep in mind that the addition of a third component can help stabilize the API in the multicomponent complex.

## Figures and Tables

**Table 1:** Some pharmaceutical products that have been approved and marketed contain CDs to minimize drug degradation (partial list).

Drug	Trade Name	Administration Route	Company
αCD			
Alprostadil (PGE1)	Caverject Prostavasin	injection injection	Pfizer Sidus
Cefotiam-hexetil	Pansporin T	oral	Takeda
βCD			
Aceclofenac	Aceclofenac-B-Cyclodextrin	oral	Taj Pharmaceuticals India
Cholecalciferol Glucagon	Vitamin D3 Baqsimi	oral nasal	Natures Aid, U.K. Eli Lilly
γCD			
Benzoyl peroxide	Nujevi Acne	dermal	Nujevi
HPβCD			
Indometacin	Indocid	ocular	Chauvin
Hydrocortisone	Dexocort	buccal	Actavis
SBEβCD			
Posaconazole	Noxafil	injection	Merck Sharp & Dohme
Voriconazole	Vfend	injection	Pfizer

**Table 2:** List of drugs complexed with various CDs and their impact on drug stability.

API	CD Used	Effect Observed	Ref.
*Clostridium difficile* Toxoid A V antigen Fibroblast growth factor 10	αCD βCD HPβCD SBEβCD γCD	inhibit protein aggregation	[19]
Human growth hormone	αCD, HPβCD, SBEβCD, Sulfated βCD, Monoglycosyl-βCD, Monomaltosyl-βCD, Monoacetyl-βCD, γCD	inhibit protein aggregation	[20]
IgG	βCD, HPβCD	inhibit protein aggregation	[21]
Glucagon	γCD	chemical and physical stability improved	[22]
Insulin glargine	SBEβCD	enzymatic degradation at the injection site reduced	[23]
Z-ligustilide	HPβCD	photostability improved	[24]
Resveratrol	SBEβCD	degradation kinetics in biological matrices inhibited	[25]
	HPβCD	stability improved	[26]
	multicomponent: HPβCD and hyaluronic acid	improved stability dependent on the polysaccharide concentration	[26]
Oxyresveratrol	HPβCD	thermal stability increased	[27]
Quercetin	αCD, βCD	photostability improved	[28]
	HPβCD	stability improved	[26]
	multicomponent: HPβCD and hyaluronic acid	improved stability dependent of polysaccharide concentration	[26]
Rutin	βCD, HPβCD	photostability improved	[29]
Ethanol extract of Cannabis sativa	DMβCD	thermal stability increased	[30]
UV filters (oxybenzone, octocrylene, and ethylhexyl-methoxycinnamate)	βCD	photostability increased	[31]
Phenylbenzimidazole sulfonic acid	HPβCD	photostability increased	[32]
Tretinoin	βCD	photostability increased	[33]
Tetra-1,2-diethylamino substituted zinc (II) phthalocyanine	αCD, βCD, γCD	aggregation decreased	[34]
Enalapril	βCD	hydrolysis and cyclization decreased	[35,36]
Hydrocortisone	HPβCD	hydrolysis decreased	[37]
		significantly increased stability after gamma irradiation	[38]
	βCD	accelerated decomposition under alkaline conditions	[39]
Famotidine	HPβCD, CMβCD	degradation reduced under acidic conditions	[40]
		physical stability improved	[41]
	SBEβCD	destabilizing effect induced	[42]
		physical stability improved	[41]
Lansoprazole	HPβCD, βCD	stabilization effects under light, heat, and humidity exposition	[43]
Camptothecin	RDMβCD	hydrolysis decreased	[44]
Nintedanib	SBEβCD	stability in simulated intestinal fluid enhanced	[45]
Posaconazole	βCD	oxidative degradation decreased	[46]
Nicardipine	βCD, HPαCD, 2-hydroxyethyl-βCD	photoprotective effect	[47]
	γCD, MβCD, HPβCD, HPγCD	no effect on photostability	[47]
	αCD	photodegradation effect	[47]
Doxycycline hyclate	βCD	photoprotective effect	[48]
Oxytetracycline hydrochloride	βCD	degradation rate reduced only for Form III	[49]
Doxorubicin	HPβCD	photostability increased	[50]
Furosemide	multicomponent:βCD and triethanolamine	chemical degradation reduced	[51]
Ascorbic acid	HPβCD	stabilizing effect pH-dependent in solution	[52]
	multicomponent: HPβCD and triethanolamine	stability in aqueous solutions improved, photodegradation reduced	[53]
	multicomponent: γCD and polyvinyl alcohol	oxidation reduced in aqueous solutions	[54]
Dihydroartemisinin	multicomponent: HPβCD and soybean lecithin	stability in aqueous solutions improved	[55]
Benzylpenicillin	HPβCD, RMβCD	hydrolysis reduced under acidic conditions	[56]
	HPβCD	hydrolysis accelerated under neutral and basic conditions	[56]
	RMβCD	catalytic effect on hydrolysis reduced under basic solution	[56]
	γCD	catalytic effect of hydrolysis	[56]
	randomly methylated γ-CD, octakis(2,3,6-triO-methyl)-γCD	catalytic effect of hydrolysis reduced	[57]
	heptakis(2,3,6-tri-O-methyl) βCD	degradation reduced by null catalytic effect	[57]
	RMβCD, heptakis(2,6-di-O-methyl)-βCD	catalytic effect of hydrolysis reduced	[57]
β-lactam antibiotics	βCD	destabilizing effect	[58]
Cefixime	βCD	destabilizing effect	[59]
Rifampicin	γCD	destabilizing effect	[60]
	multicomponent: γCD and arginine	stabilizing effect	[60]
Norfloxacin	βCD	photostability of Form C increased	[61]
		chemical stability of Form B hydrate decreased	[62]
Omeprazole	βCD, DMβCD, HPβCD, MaβCD	hydrolysis accelerated	[63]
Prostaglandins	βCD	destabilizing effect	[64]
	DMβCD	stabilizing effect	[64]
Irbesartan Candesartan cilexetil	multicomponent: γCD and organic salts	hydrolysis increased	[65]

## Data Availability

Data sharing not applicable.
